# Histological, chemical and behavioural evidence of pedal communication in brown bears

**DOI:** 10.1038/s41598-017-01136-1

**Published:** 2017-04-21

**Authors:** Agnieszka Sergiel, Javier Naves, Piotr Kujawski, Robert Maślak, Ewa Serwa, Damián Ramos, Alberto Fernández-Gil, Eloy Revilla, Tomasz Zwijacz-Kozica, Filip Zięba, Johanna Painer, Nuria Selva

**Affiliations:** 1 Institute of Nature Conservation, Polish Academy of Sciences, Adama Mickiewicza Av. 33, 31120 Kraków, Poland; 2 Department of Conservation Biology, Estación Biológica de Doñana CSIC, Avd. Americo Vespucio s/n, 41092 Seville, Spain; 3 Wrocław Research Centre EIT+, Stabłowicka 147, 54006 Wrocław, Poland; 4 Department of Evolutionary Biology and Conservation of Vertebrates, Institute of Environmental Biology, University of Wrocław, Sienkiewicza Str. 21, 50335 Wrocław, Poland; 5 Consejería de Desarrollo Rural y Recursos Naturales, Gobierno del Principado de Asturias, C/Coronel Aranda, 2 - Planta 3ª, 33005 Oviedo, Spain; 6 Tatra National Park, Kuźnice 1, 34500 Zakopane, Poland; 7 Department of Integrative Biology and Evolution, University of Veterinary Medicine, Savoyenstraße 1, 1160 Vienna, Austria

## Abstract

Most mammals rely upon scent for intraspecific communication. As most bear species have large home ranges and are non-territorial, scent deposit while walking could be an effective way to communicate with conspecifics. Here, we investigate the existence of pedal glands in brown bears and their role in chemical communication from a histological, biochemical and behavioural perspective. We found eccrine glands in footpads, and prominent apocrine and sebaceous glands in the interdigital, metacarpal and metatarsal skin sections. Pedal scent contained 26 compounds including carboxylic acids, important constituents of mammalian secretions. Six of these compounds were exclusive for males. Finally, we describe a specific marking gait recorded in the field, mostly performed by males. Our study supports the existence of chemical communication through pedal marking in brown bears and suggests sex-coding potential of pedal scent.

## Introduction

Chemical signaling is a widespread mode of communication amongst the vast majority of organisms, both terrestrial and aquatic^1^. Among mammals, odours play a key role in communication, with chemical signaling used to inform about identity, sex, territorial borders, social status, reproductive state or group membership^2, 3^. Apart from the prevalent chemical features of urine and faeces, mammal skin secretions offer a great potential for chemical communication, with many species showing specialized scent glands that are the main source of secretions in scent marking^4–7^. Well known is the use of scent marking at communal latrines by badgers (*Meles meles*) and other mustelid species, which have anal glands used for specific squat-marking^8^. Marking with subcaudal, chin, foot and ventral glands, and through scratching and rolling has also been recorded^7, 8^. The understanding of the mechanisms behind the detection of scents and how the scent stimuli is reconstructed into scent maps used by animals in their environment has greatly advanced, but the world of odours and the way they are perceived still require a great deal more research and experimentation^9^.

The skin of mammals typically presents three types of glandular structures: holocrine sebaceous glands, apocrine sweat and eccrine sweat glands^4, 10^. Sebaceous glands are evenly distributed over the body. They coat hair with sebum as it grows and are usually anatomically near an apocrine gland^11^. Apocrine sweat glands, opening to follicles, are primarily observed in hairy skin^12^, while eccrine sweat glands secreting directly to skin surface via pores tend to be confined to specific regions, such as carnivores’ footpads^5^ or friction surfaces of hands, feet and tails of prosimians, monkeys and apes^11^. These basic types of glands may associate in more complex structures (e.g. sacs) often located in specific areas of the skin. Finally, the presence of mammalian scent glands is often linked to typical behavioural patterns of scent marking^4–6, 13^. Therefore an in-depth examination of the existence of scent glands is the first step to disentangle chemical communication.

Most areas containing sebaceous and sweat glands can be involved in scent production and synthesize different odoriferous molecules^14^. The secretions of these glands can be ultimately affected by bacterial fermentation, which has been shown to be involved in odor production^15, 16^. The relevant molecules are fixed in the secretion of the sebaceous glands to be slowly released to the environment^14^. The compounds found in interdigital pockets, characterized by enlarged sebaceous glands with a few apocrine glands, as occurs in ungulates, include ketones, esters, arenes, alkanes, aliphatic acids and pyrolles associated with lipid secretions^10, 17^, but also background volatiles that may remain in the environment for several hours or longer if secreted in a lipid matrix^10^. For instance, brachial glands in ring-tailed lemurs (*Lemur catta*) are characterized by sebaceous and apocrine glands that secret a greasy mixture comprising mainly lipids as fixative and waterproofing agents that fix more volatile components, thereby increasing the longevity of scent marks^13^. The secretory products of the eccrine glands in the footpads of domestic cats (*Felis* catus) and dogs (*Canis familiaris*), raccoons (*Procyon lotor*) and wolves (*Canis lupus*) exhibit high amounts of glycoconjugates, which are of importance for territorial scent marking activities, tracking abilities and orientation^5^.

The specific position of glands on the body surface seems to respond to its particular behavioural use^4, 5, 13, 18^. Facial glands are well documented for a variety of chiropteran species that use them to face-mark roosts and females within social groups^18^ and for mustelids, that among other marking behaviours, display also marking with chin glands^7^. Pockets of sebaceous glands on the axillary surface of shoulders and apocrine gland fields on wrists in lemurs^13^ are used in shoulder-rubbing and wrist-marking behaviours to advertise social rank, reproductive state, and to mark territories and mediate inter-group spacing. Scent marking glands in the forefoot of aardwolf (*Proteles cristatus*) are activated with rubbing the knuckles on the ground^4^. Similar ground scratching with feet for chemical marking is also observed in brown (*Hyaena brunnea*) and spotted (*Crocuta crocuta*) hyaenas^19^, mustelids^7, 8^ and canids^20^. Footpads possess a remarkable sensory apparatus, and additionally, the biological function of their eccrine glands is to improve the frictional capacities of the paw and to leave scent marks^21^.

Ursids have large home ranges and a solitary and non-territorial life style. Thus, they must rely on effective modes to communicate with conspecifics, which still are largely unexplored. Only recently, some studies have dealt with signaling behaviours in bears, mostly related to tree marking^22–26^. Tree rubbing by brown (*Ursus arctos*) and black bears (*Ursus americanus*) have been proposed as a main signal to communicate dominance^23^. The secretions from anal glands^27^ and sacs^6^ have been shown to code for sex-related cues in at least two bear species. The presence of sweat glands recently found in polar bear (*Ursus maritimus*) paws suggest that scent marks can be also passively deposited while walking and can originate from pedal glands, urine, or a combination of these two^28^. Therefore, pedal scent marking may be a widespread chemical signaling among ursids, which is still poorly understood and has not been systematically studied in an integrated manner from a histological, biochemical and behavioural perspective.

In this study we investigate the existence of pedal scent communication in brown bears, from the presence and histology of pedal glands, through biochemical composition of their secretions, to the description of behaviours associated with pedal scent marking. We first sought evidence that brown bears possess pedal glands by histological examination of interdigital, metacarpal, metatarsal and footpad skin, and compared the findings to skin sections of other parts of the body. Second, we identified the chemical compounds associated with pedal scent glands with gas chromatography techniques. Finally, we provided field evidence of behaviours related with pedal marking by brown bears of different age and sex classes throughout the year in a long-lasting marking site.

## Results

### Histological examination of pedal and control skin

**Figure 1:**
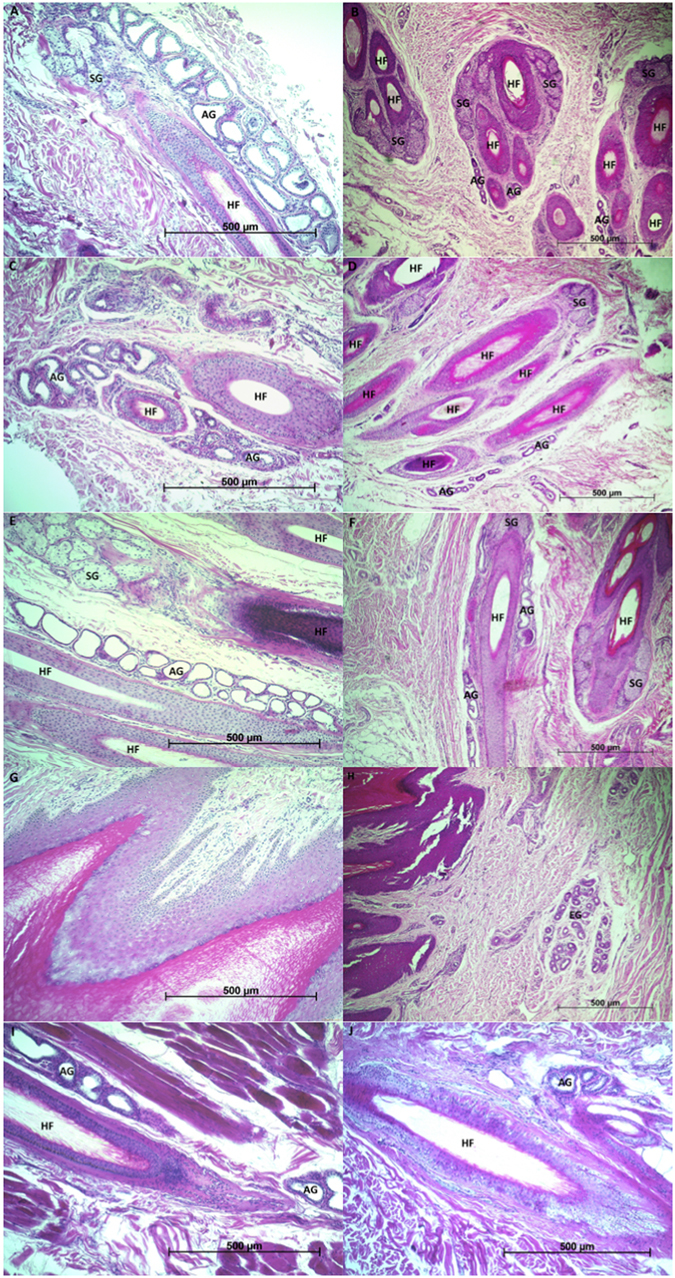
Histological sections of interdigital skin of front paw in adult male (**A**), interdigital skin of front paw in yearling male (**B**), interdigital skin of hind paw in adult male (**C**), interdigital skin of hind paw in yearling male (**D**), ventral metatarsal skin in adult male (**E**), ventral metatarsal skin in yearling male (**F**), front footpad in adult male (**G**), front footpad in yearling male (**H**), and control lip skin (**I**) and control left shoulder skin (**J**) in adult male brown bear (*Ursus arctos*). In interdigital and metatarsal regions (**A**,**B**,**C**,**D**,**E**,**F**) there are hair follicles (HF) visible with apparently more profuse and prominent apocrine sweat glands (AG) in adult male (**A**,**C** and **E**). In sections of control skin of the adult male (**I** and **J**), apocrine sweat glands appear less profuse than in sections of the focus areas of the paws. Sections of footpads (**G** and **H**) show the stratified squamous keratinized epithelium of the pad with very thick striatum corneum (the darkest shade of stain), and eccrine glands (EG) deeper in the dermis of yearling male (**H**). Stain: hematoxylin and eosin. Magnification: x100. SG – sebaceous gland.

We did not observe any macroscopically apparent glandular structures in the sampled regions of the two bears examined at necropsies. Microscopically, the ventral interdigital, metacarpal and metatarsal skin sections of the adult male presented prominent apocrine sweat glands and holocrine sebaceous glands in association with hair follicles (Fig. 1). Glands present in interdigital and metacarpal/metatarsal skin were more prominent than those present in the control sections of the lip and shoulder. The yearling male also presented apocrine glands in the interdigital and metacarpal/metatarsal skin sections (Fig. 1B,D and E), but they were less prominent compared to those of the same skin regions of the adult male in terms of the number of segments per gland (see comparison of morphological features in the Supplementary Table 1). We observed also a small number of eccrine sweat glands in the deep dermis and subcutaneous tissue of the footpads of both studied individuals.

### Chemical compounds of the pedal scent

**Figure 2:**
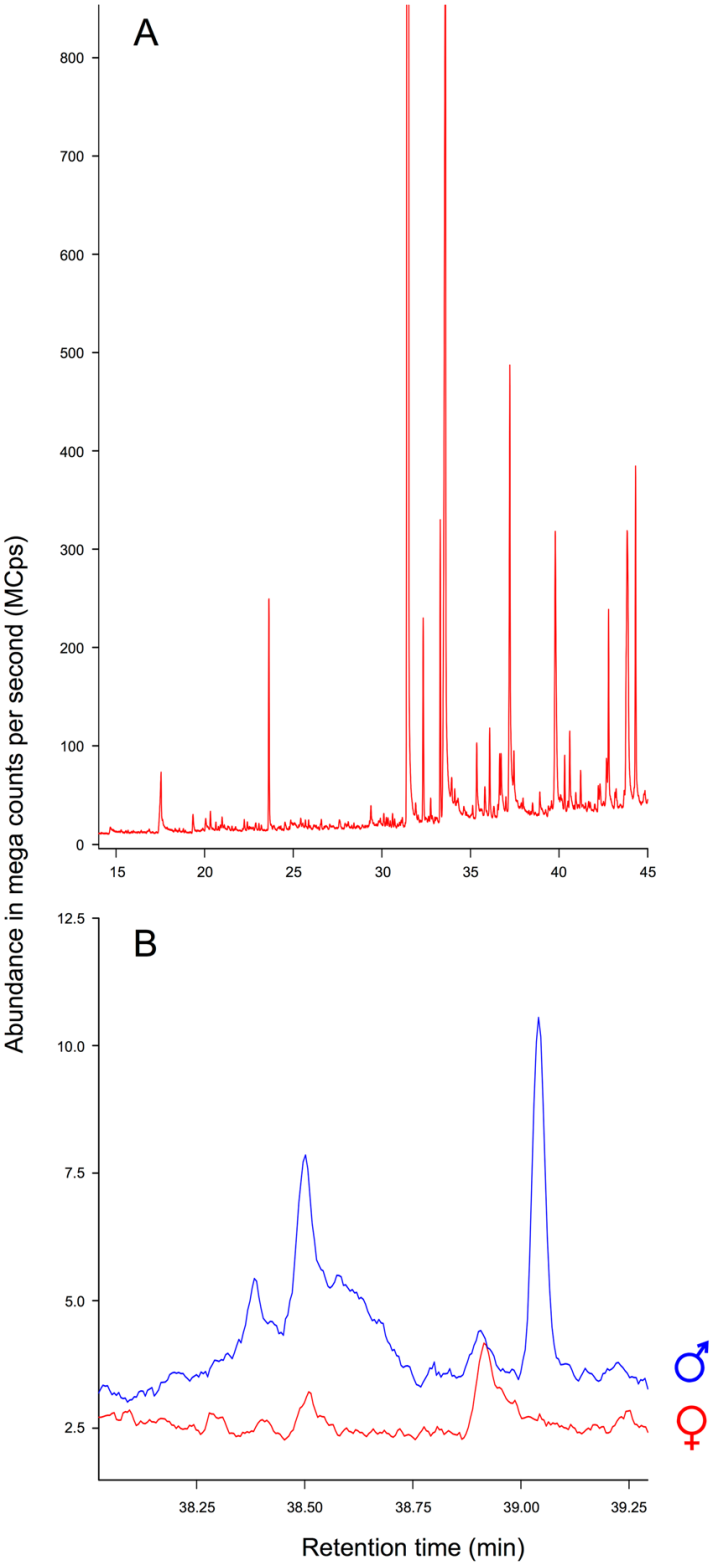
Typical gas chromatogram (**A**) of the pedal swabs of a brown bear (*Ursus arctos*), and (**B**) magnified fragments of chromatograms of an adult male (blue) and an adult female (red) brown bear with observed certain differences in profile peaks with retention time 38.4 and 39.04 minutes. Retention times correspond with those given in Table 1. The x-axis is the retention time in minutes and the y-axis is the abundance in mega counts per second (MCps) scale.

We identified twenty distinct compounds in swabs of sampled free-ranging bears, occurring in all samples irrespectively from the paws they were swabbed from. Six additional compounds were found only in the samples of all adult males (N = 8) but not in the samples of adult females and the yearling male (N = 12 and N = 4, respectively; Fig. 2 and Table 1). The compounds found in all samples of the yearling male (N = 4) corresponded with those of the female profile.

**Table 1:** Retention times and molecular masses of compounds tentatively identified in pedal swabs of brown bear (*Ursus arctos*; 1 – present in all samples, 0 – absent in all samples).

Retention time (min)	Adult females’ samples (N = 12)	Yearling male samples (N = 4)	Adult males’ samples (N = 8)	Tentatively identified compounds	Molecular mass	CAS no	Chemical characteristics
8.99	1	1	1	Hexanoic acid	116	142-62-1	Carboxylic acid, skin-borne
14.74	0	0	1	Octanoid acid	144	124-07-2	Carboxylic acid, skin-borne
17.54	1	1	1	Nonanoic acid	158	112-05-0	Carboxylic acid, skin-borne
19.34	1	1	1	Unknown	—	n/a	—
20.06	1	1	1	n-Decanoid acid	172	334-48-5	Saturated fatty acid, skin-borne
20.32	1	1	1	Unknown	—	n/a	—
20.64	1	1	1	Unknown	—	n/a	—
20.89	1	1	1	Vanillin	152	121-33-5	—
21.1	1	1	1	Unknown	—	n/a	—
21.54	1	1	1	Unknown	—	n/a	—
21.75	1	1	1	Unknown	—	n/a	—
22.88	1	1	1	Unknown	—	n/a	—
25.42	1	1	1	Unknown	—	n/a	—
27.6	1	1	1	Unknown	—	n/a	—
27.9	1	1	1	Cyclohexanone, 5-octyl-3,3,5-trimethyl	252	129126-26-7	Ketone of animal origin
28.42	1	1	1	Unknown	—	n/a	—
29.38	1	1	1	Tetradecanoid acid	228	544-63-8	Saturated fatty acid, skin-borne
30.47	1	1	1	Phthalic acid, dodecyl 2-isopropooxyphenyl ester	468	n/a	Aromatic carboxylic acid, organic
31.9	1	1	1	1-Hexadecanol	242	36653-82-4	Fatty alcohol, skin-borne wax
33.11	0	0	1	Thunbergene	272	1898-13-1	Cembrenoid, diterpene, might be both animal and plant origin
34.65	0	0	1	Nerolidol	222	7212-44-4	Sesquiterpene of plant origin
35.12	1	1	1	Thunbergol	290	25269-17-4	Diterpene of animal or plant origin
36.32	0	0	1	Unknown	—	n/a	—
36.31	1	1	1	Unknown	—	n/a	—
38.54	0	0	1	Unknown	—	n/a	—
39.04	0	0	1	Dehydroabietate	284	13601-88-2	Resin acid

The compounds identified in the study (see Table 1 for the retention times and molecular masses of all compounds observed), beside those originating from the environment, included skin-borne hexanoic (caproic), nonanoic (pelargonic), n-decanoid and tetradecanoid (myristic) acids, 1-hexadecanol, and cyclohexanone. The group of compounds that were detected only in adult males included skin-borne octanoid (caprylic) acid, thunbergene, two compounds that could not be identified due to low signal-to-noise ratio, and two compounds of potential plant origin. Thunbergene belongs to cembrenoid diterpens, which can be both of animal and plant origin.

### Pedal marking behaviour

During our study period, from January 2012 through February 2015, we detected bear activity in 18% of the camera trap days (N = 752 days). Brown bear behaviour was recorded on 232 videos and bear activity at the study site occurred in all months, except for January. At least fifteen different bears were individually recognizable by means of shapes and colours of hair marks on their head and neck. The individuals recorded included seemingly solitary individuals, adult males (either alone or in the company of adult females), adult females with offspring (cubs either first or second year), sub-adult bears (such as lone individuals or groups of siblings, recently weaned), and bears of unknown sex and age.

**Figure 3:**
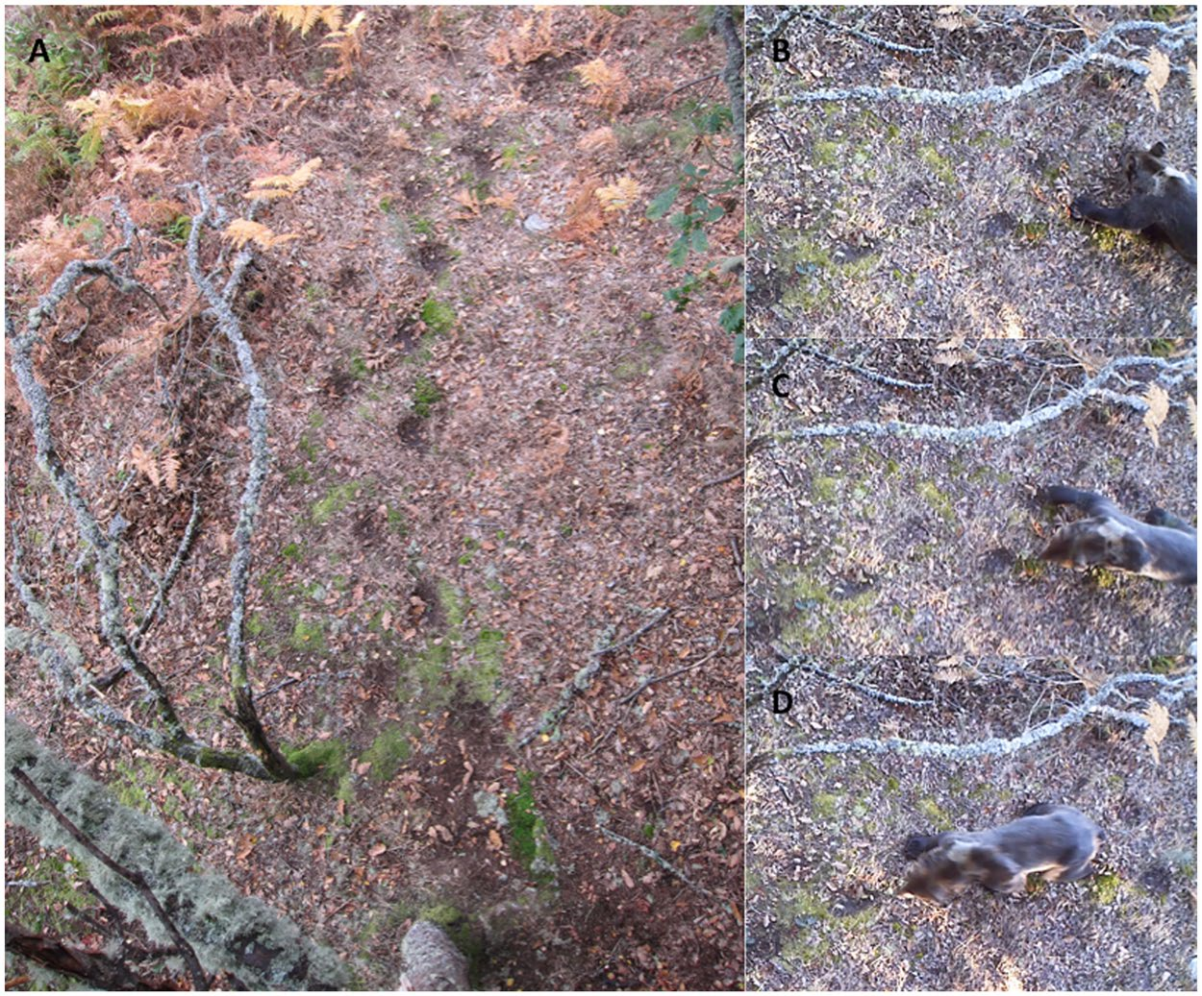
Snapshot from the camera in the studied marking site with visible depressions from previous pedal marking (**A)** and a brown bear individual stretching to match the marks (**B**–**D**).

Pedal marking was recorded in 81 videos (35%), mostly performed by adult males (96% of the recorded pedal marking) and in a few instances by sub-adult bears of unknown sex (4% of the pedal marking events). Pedal marking behaviour is characterized by individuals using a characteristic gait while walking, carefully stepping and twisting their feet in depressions made in the ground (Fig. 3) by the repeated use of the marking site along time. Seasonally, pedal marking was observed in all months between March and November, but peaked during the mating season (April-June, 54% of the recorded videos with pedal marking). We also observed sniffing of pedal marks in 38 videos (16%). Mostly adult males performed sniffing (58% of the videos with sniffing behaviour), but other sex and age classes, including adult females, were also sniffing the marks. The sniffing of pedal marks was recorded between March and December, and distributed throughout the year.

## Discussion

**Figure 4:**
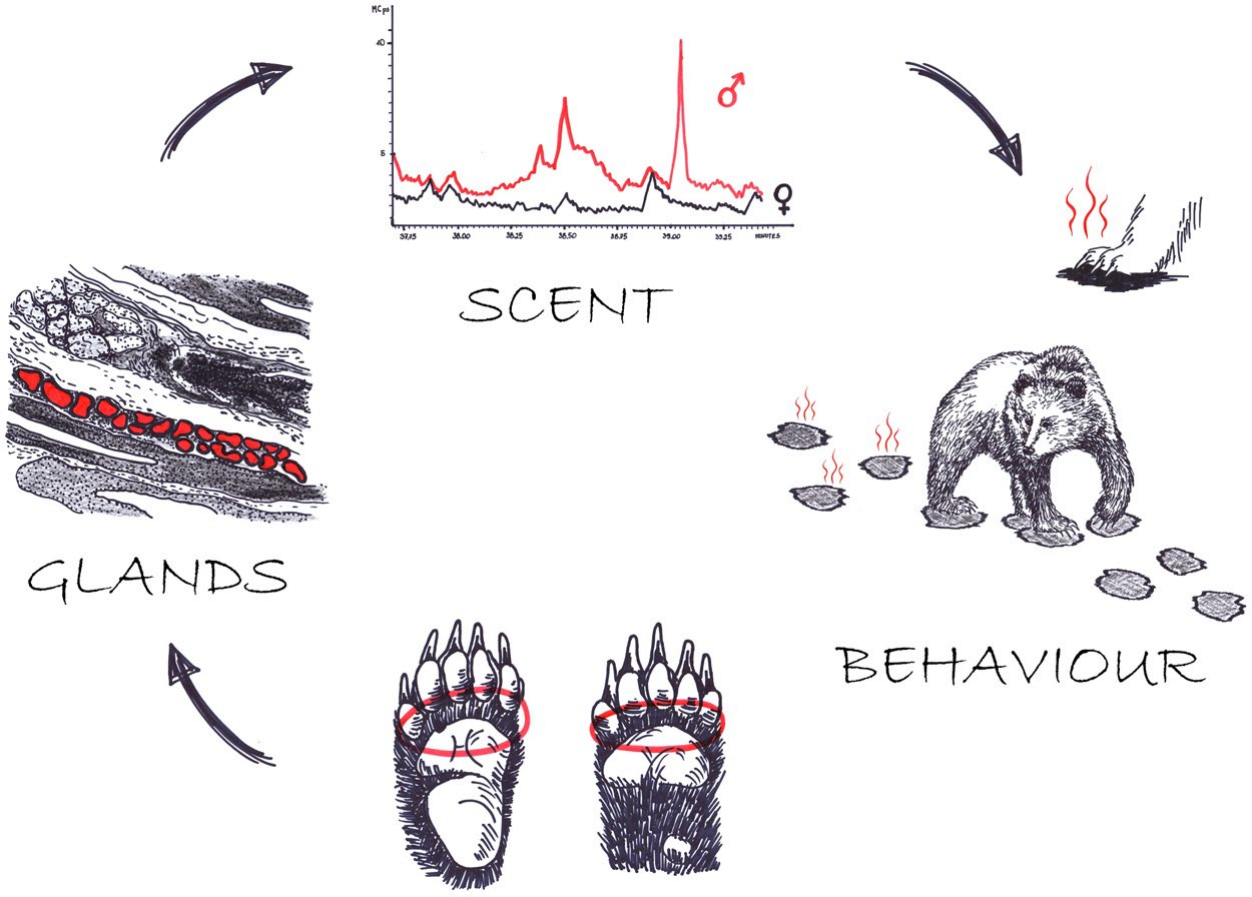
Graphical abstract. The study demonstrates that brown bears do have pedal glands that produce specific and sexually dimorphic scent, and display behavioural patterns related to pedal marking. Drawings credits Katarzyna Chrząścik.

Our histological and biochemical examination of bear paws, coupled with behavioral observations, demonstrated the existence of relevant forms of chemical communication in brown bears. We found that brown bears do have pedal glands that produce specific scents, and showed field evidence for behavioural patterns related to pedal marking. To our knowledge, this is the first histological, chemical and behavioural joint evidence description of this communication mode in brown bears (Fig. 4).

Apocrine glands, which contribute to the secretions of most mammals^15^, form the most prominent feature of the sampled regions of pedal skin. These structural glandular components support the existence of pedal scent marking in brown bears. Apocrine glands, observed primarily in hairy skin and opening to follicles^12^, were identified in interdigital, metatarsal and metacarpal skin, connected to follicles and adjacent to sebaceous glands comprising the bulk of secretory elements, while eccrine glands appeared to be confined to footpads, as occurs in other carnivores^5^. It has been suggested, based on studies of chemical components of scent in ungulates^10^, primates^13^ and carnivores^4^, that areas of glandular skin are composed of apocrine and sebaceous glands forming a functional unit^4^ with hair-erecting muscles acting to force the secretion out of the ducts and hair follicles^10^.

Mammalian scent glands secretions typically contain a wide range of compounds, including aldehydes, alcohols, ketones, esters, sterols, acids, proteins and aromatics^29^. Some of the chemical compounds identified in this study belong to a group of carboxylic acids, important constituents of skin-borne body odours and vaginal secretions in mammals (e.g. in human and nonhuman primates)^30^, and thus presumably of biological significance for the species. Cyclohexanone, suggested to be a characteristic scent-marking compound for large mammals^31^, appeared in all sampled bears. Notably we found cembrenoid exclusively in the pedal scent of adult males. Cembrenoids have been isolated from soft corals (*Nephthea* spp.) and are also found in higher plants. Nevertheless, they have been identified among trail pheromones in Australian subterranean termite (*Nasutitermes exitiosus*) and recognition pheromones in the pharaoh ant (*Monomorium pharaonis*)^32^. Cembrene A was confirmed in Chinese alligator’s (*Alligator sinensis*) paracloacal glandular secretion^33^ that contains pheromones thought to play a role in nesting and/or mating behaviour^34^. Cembrenoid presence in the scent of male brown bears suggests that chemical communication in the species may have evolved a range of communicative functions, both inter- and intrasexually. It also may be associated with higher tree marking activity in adult males than females^24^.

Only one chemical analysis has been conducted on brown bear glandular secretions. Rosell *et al*.^6^ showed that brown bear anal gland secretion contains a high number of compounds with high molecular mass (above 300 g/mol), indicating its possible use in long-lasting scent marks. The pedal scent compounds identified in our study were in a range of molecular mass from 116 to 468 g/mol, with the majority below 300 g/mol. Volatility usually decreases with higher molecular mass^35^, although some compounds with relatively high molecular masses can still be sufficiently volatile to be used in chemical communication^36^. With increased size or polarity, the evaporation rate declines, and the signal can be emitted for a longer period^35^. Cyclohexanone, identified among pedal scent compounds in the study, has a slow volatilization period of hours and is responsible for a relatively long lasting signal^31^. Giant pandas (*Aiuloropoda melanoleuca*) were still able to detect and respond to secretions about 120 days old^37^. The durability of chemical signals can be modulated by using secretions separately or by mixing the more lipid secretion of sebaceous glands with more volatile and water-soluble secretion of apocrine glands^13^. Interdigital, metacarpal and metatarsal regions sampled in our study contained apocrine glands and sebaceous glands associated with hair follicles, and additionally eccrine glands in footpads, that would explain possible scent deposits while walking and its potential to last in the environment due to mixed secretions.

It appears difficult to determine if the pedal scent origin is of glandular secretions only or has an additional effect of urine^28^, with some additional volatile compounds potentially arising from microbial decomposition of urinary compounds and glandular secretions. Some ungulate species urinate onto their tarsal glands in a behaviour called rub-urination. Urine becomes trapped by hair, facilitating the mixing of glandular secretions and urine, possibly producing unique odours^15^. Brown bears, particularly males, have been observed urinating while tree-marking^23, 38^, and while walking^23^. Nevertheless, we did not detect urine-derived compounds, such as urea, *N*,*N*-dimethyl urea, tetramethylthiourea, trimethyl urea, or cyanuric acid^29^.

During pedal marking bears show a characteristic gait. This behaviour seems often linked with individuals approaching rubbing trees, as shown by the data collected in our study field site. Bears walk carefully stepping and twisting their feet in the previous marks left by itself or other individuals, generating a marked trail which can be used for years and by several bears (consecutively, not simultaneously) as in our study site (Fig. 3). Therefore, pedal marking can also be associated with other communication channels, such as visual. In giant pandas, foot scraping the substrate with the hind paws takes place in the presence of male odours more than female odours, seemingly linked to intrasexual competition, and may serve as a visual as well as olfactory signal^39^. This intrasexual aspect is in agreement with our finding that mostly males are marking with pedal glands. Clapham *et al*.^25^ observed pedal marking in brown bears only in adult males and across seasons, and inferred its function to distribute pedal scents if the species had modified apocrine glands similar to polar bears^28^. The so-called stomp walking and sumo strutting have been also defined as parts of stereotypic behaviours shown during inter-individual or bear-human encounters^38^. Lloyd^40^ described ground marks in American black and brown bears associated with disturbed capture sites, fishing sites, daybeds and marking trees, but also found a few cases where they were not associated with any other bear sign. Furthermore, the author suggests that in some situations, and in the absence of the usual communication signals, ground marking functions as visual and tactile communication mode, and may substitute for olfactory signals. Behavioural odour assays tested recently in polar bears^28^, showed bears spent more time investigating pedal scent from the opposite sex, what would strongly suggests its role in olfactory communication with potential coding for sex. The significance of tracks scent-marked for sex recognition in this species was hypothesized by Stirling and Derocher in 90 s^41^. The differences we found in chemical compounds between samples of adult males and females support that interpretation.

The secretory capacity of sweat glands may be related to the age of the animal^42^. The scent-sampled yearling male appeared matching adult females in its chemical profile. Also the morphological features of its glands appeared less prominent than those found in the adult male, what seems supporting a hormone-regulated gland activity associated with puberty^43^.

## Conclusions

We show that brown bears have apocrine and eccrine sweat, and holocrine sebaceous glands in their paws, and characterize the chemical signals in brown bear pedal scent. Chemical compounds of adult bears appeared sexually dimorphic, suggesting sex-coding potential of pedal scent. This evidence is supported by our field data, which show that males displayed pedal marking behaviour more frequently, particularly in the mating season, in a marking site that was used for several years and by a number of bears. Our study reveals another species of solitary carnivore that communicates through its feet and that this is an important way of information transfer. The findings advance the knowledge of the communication modes in ursids and may promote research of pedal scent communication in wide-ranging species.

## Methods

### Histological examination of skin samples

To investigate the ability of brown bears to produce pedal chemical signals, first we performed a histological examination of the pedal skin of two individuals (one captive adult male and one yearling male from the wild). All four paws of the captive adult male brown bear (euthanized due to an advanced degenerative joint disease) were carefully examined for glandular tissue. We collected twelve skin biopsies (square excisions of at least 10 × 10 mm collected using scalpel and including full thickness of skin and subcutaneous fat, wherever possible) from the dorsal and ventral interdigital region, the ventral metacarpal and ventral metatarsal regions, and footpads. Additionally, we collected a total of four control biopsies from the left shoulder (N = 2) and the lip (N = 2) for comparison. From the yearling male found dead as a result of intraspecific killing, only two paws (one front and one hind) were available for biopsies. We collected six skin biopsies from the dorsal and ventral interdigital region, the ventral metacarpal and ventral metatarsal regions, and footpads. All skin samples were immediately fixed in 10% neutral formalin, processed routinely for histology, embedded in paraffin and sectioned at 7 microns. Sections were stained with hematoxylin and eosin.

The biopsies were not even by means of surface, size and depth; for instance, interdigital skin has smaller surface and less profuse layers than pads or lip. We selected four to 14 sections from the sampled regions and counted the number of segments of particular glands, the number of glands associated with hair follicles, and the ratio of follicles with apocrine glands to follicles without these glands. To compare particular sections of sampled regions, all pictures were taken with the same magnification (x100). Sections were examined with a ZEISS Axioskop and pictured with ZEISS AxioCam ERc 5s camera using ZEISS AxioVision Release 4.8.2 software (ZEISS, Goettingen, Germany).

### Collection and analysis of scent samples

We performed scent collection on six wild brown bears captured and immobilized in the northern Carpathians (S Poland). Bears were captured in a modified culvert trap or Aldrich traps and immobilized following the biomedical protocols by Arnemo *et al*.^44^ in 2014–2016. All capture and handling procedures were approved by the Polish authorities and the Ethical Committee (see ethics statement).

The GC/MC analyses were carried out by gas chromatography laboratory at the Wrocław Research Centre EIT+, Wrocław, Poland. First of all, we conducted a pilot study to test the performance of two types of swabs for scent collection (with plastic and wooden core, respectively), the effectiveness of different solvents (methanol, methanol:toluene solution 1:3, and hexane) and the effectiveness of extraction with hexane after two and 24 hours. The pilot study revealed that the swabs with a wooden core, the hexane solution and the 2-hour extraction time were most appropriate for sampling (see Supplementary information for the details).

Twenty-four pedal scent samples were collected from six bears (two adult males, one yearling male, and three adult females). Samples were collected using medical-grade sterile cotton swabs for microbial sampling with wooden core (C050, HAGMED, Rawa, Poland). Feet were firmly rubbed several times (between three and five times each area) by the tip of the swab against dorsal and ventral interdigital, ventral metacarpal and metatarsal regions. To avoid potential contamination from human scent, single-use nitrile gloves were used during collection. Each swab was then cut 2 cm above the cotton, placed separately into 4 mL glass vials (ND 13, Bionovo, Legnica, Poland) with teflon PTFE-butyl-lined cap (ND 13, Shore hardness scale 55° A, Bionovo, Legnica, Poland), and immediately put in a field fridge with ice for transport. All samples were stored at −20 °C within 3 hours from collection and kept frozen until analysis.

In order to perform the extraction for subsequent gas chromatography/mass spectrometry analyses, each swab was transferred to a 2 ml glass vial and 1 mL of hexane was added. The sample was then vortexed for 2 hours, cleaned by 20 ul PFTE membrane filter and transferred into gas chromatography vial. The analyses were performed in a Bruker 456 gas chromatograph connected to the Triple Quad Mass Spectrometer SCION (Bruker, Poznan, Poland) and equipped with Restek-5MS column (30 m × 0.25 mm ID × 0.250 µm film thickness; Anchem, Warsaw, Poland). Constant flow (1 ml/min) of helium was set and the spiltless technique was applied. Injector temperature was set at 250 °C and the single taper 4 mm liner with wool was used. The 1 µl of particle-free solution was injected into GC-MS system. The initial temperature was set at 50 °C with a 2-min hold, and then increased by 5 °C per minute to 260 °C (with a 2-min hold at 260 °C). Solvent delay was set to 5 min. Unused (blank) swabs were subjected to the same sampling protocol to test for background compounds in the swabs and used apparatuses. Compounds were tentatively identified by matching their retention time and mass spectra with structures available in the NIST 11 library^45^. In order to improve detectability and identification, compound data were deconvoluted and processed through Automated Mass Spectrometry Deconvolution and Identification System (AMDIS) detection tool. The mass spectra of peaks that were not found in the library or had a match score lower than 0.8 are reported as unknown.

### Field observations of brown bear behaviour

Pedal marking behaviour of wild brown bears, as well as related field signs, were observed in the Cantabrian Mountains (NW Spain) during field work in several areas and for several years. A single spot located in a deciduous oak forest (*Quercus* spp.), where pedal marking behaviour was previously noted, has been regularly monitored with a video-recording camera trap (Bushnell Trophy Camera, model 119466, Barcelona, Spain) for three years (from January 2012 until February 2015). The camera was placed over a rubbing tree (zenithal position), and triggered by the bears’ movements when approached. Each video record lasted one minute and was set at 10-second intervals. We considered an instance of “pedal marking behaviour” when a bear was walking by placing its feet in the exact spot of any of the existing ground marks (visible as depressions), while twisting vigorously each foot during the pace. We considered an instance of “sniffing of pedal marks” when the focal individual was lowering its head, placing its nose over the tracks and sniffing them. We recorded the date for each observation of these behaviours. Whenever possible, age and sex classes were determined from videos through the observation of genitals, body shape and size, and presence of accompanying individuals.

### Ethics

The procedures of animal captures and handling in this study fully complied with Polish legal requirements and were approved by the Polish authorities (General Directorate for Environmental Protection in Warsaw: permit #DOPOZ.6401.08.2.2013.ls, #DOP-OZ.6401.08.2.2013.ls.1, and #DZP-WG.6401.08.8.2014.JRO) and the Ethical Committee (I Local Ethical Committee in Krakow: permit #21/2013 and #101/2014).

## Electronic supplementary material

Supplementary information
