# Supplementary material for: Histological, chemical and behavioural evidence of pedal communication in brown bears
Source: Sci Rep. 2017 Apr 21;7:1052. doi: 10.1038/s41598-017-01136-1 (PMC5430784; doi:10.1038/s41598-017-01136-1)
Supplement: Supplementary file 1 — Supplementary information [file 41598_2017_1136_MOESM1_ESM.pdf]

Histological, chemical and behavioural evidence of pedal communication in brown bears

Agnieszka Sergiel[1*], Javier Naves[2], Piotr Kujawski[3], Robert Maślak[4], Ewa Serwa[4], Damián Ramos[5], Alberto Fernández-Gil[2], Eloy Revilla[2], Tomasz Zwijacz-Kozica[6], Filip Zięba[6], Johanna Painer[7] & Nuria Selva[1]

[1]Institute of Nature Conservation, Polish Academy of Sciences, Adama Mickiewicza Av. 33, 31120 Kraków, Poland. [2]Department of Conservation Biology, Estación Biológica de Doñana CSIC, Avd. Americo Vespucio s/n, 41092 Seville, Spain. [3]Wrocław Research Centre EIT+, Stabłowicka 147, 54006 Wrocław, Poland. [4]Department of Evolutionary Biology and Conservation of Vertebrates, Institute of Environmental Biology, University of Wrocław, Sienkiewicza Str. 21, 50335 Wrocław, Poland. [5]Consejería de Desarrollo Rural y Recursos Naturales, Gobierno del Principado de Asturias, C/Coronel Aranda, 2 - Planta 3ª, 33005 Oviedo, Spain. [6]Tatra National Park, Kuźnice 1, 34500 Zakopane, Poland. [7]Department of Integrative Biology and Evolution, University of Veterinary Medicine, Savoyenstraße 1, 1160 Vienna, Austria.

*Correspondence and requests for materials should be addressed to A.S. (sergiel@iop.krakow.pl)

Supplementary Information:

1. Supplementary Text
2. Supplementary Figures 1 to 3
3. Supplementary Table

1. Supplementary Text

Methods

We conducted a pilot study to test the performance of two types of swabs for scent collection (with plastic and wooden core, respectively), the effectiveness of different solvents (methanol, methanol:toluene solution 1:3, and hexane) and the effectiveness of extraction with hexane after two and 24 hours.

Experiment on swabs and solvents

First, we performed the experiment on the plastic swabs using toluene:methanol solution 1:3, and the extraction was impossible due to toluene dissolving the core. The next solvent used to extract swabs with plastic core was methanol. Derived extracts were analyzed using gas chromatography. We compared the chromatograms of the sterile plastic core, the sterile cotton after being removed from the core, and an actual sample from the field. We used each part of the sterile plastic swab separately to check for potential reactions of the solvent with its substance due to previous experience with toluene:methanol solution 1:3. Sterile plastic core and cotton used separately in this experiment showed strong background affecting the actual samples assays (see Supplementary Fig. 1 for a comparison).

As a next step in the pilot study, we performed a trial on the swabs with wooden core and the extraction with different solvents, namely, methanol and hexane (excluding toluene:methanol solution 1:3). We divided one sample into two parts and extracted them separately, one using methanol, and the other one using hexane. Signals obtained from the extract with hexane appeared more intense (see Supplementary Fig. 2).

In a summary, this part of the pilot study with experiments on extraction with different solvents and on different types of swabs, revealed methanol the least effective with extractions, the methanol:toluene solution dissolving plastic core, and hexane to be the most

effective (Supplementary Fig. 2). Plastic core in swabs appeared to react with solvents and to give background signals interfering with the sample signals; therefore we do not recommend its use for scent sampling. Other solvents and extraction techniques should be tested and proved effective before using them for investigations on scent compounds. Moreover, it is essential to consider preparation procedure on swabs, as “sterile” does not mean chemically clean. Therefore, pre-extraction on blank swabs in different solvents (polar and non-polar) is worthwhile and strongly recommended as a first step; then the samples can be collected subsequently on chemically clean swabs.

Experiment on extraction time

We performed a trial on different times of extraction with the use of hexane and compared the chromatograms after 2 and 24 hours of extraction. We used one sample and analyzed an aliquot of extract after 2 hours and then another one after 24 hours of extraction. The experiment revealed that the 2h extraction gave the same range of signals as the 24h extraction (see Supplementary Fig. 3). Therefore, the shorter extraction time was used further on in the study.

Results

See supplementary Table 1 for comparison of morphological features of apocrine sweat glands in interdigital, metacarpal and metatarsal regions of the sampled adult and yearling brown bear (*Ursus arctos*) males, and control skin sections of adult male.

2. Supplementary Figures 1 to 3

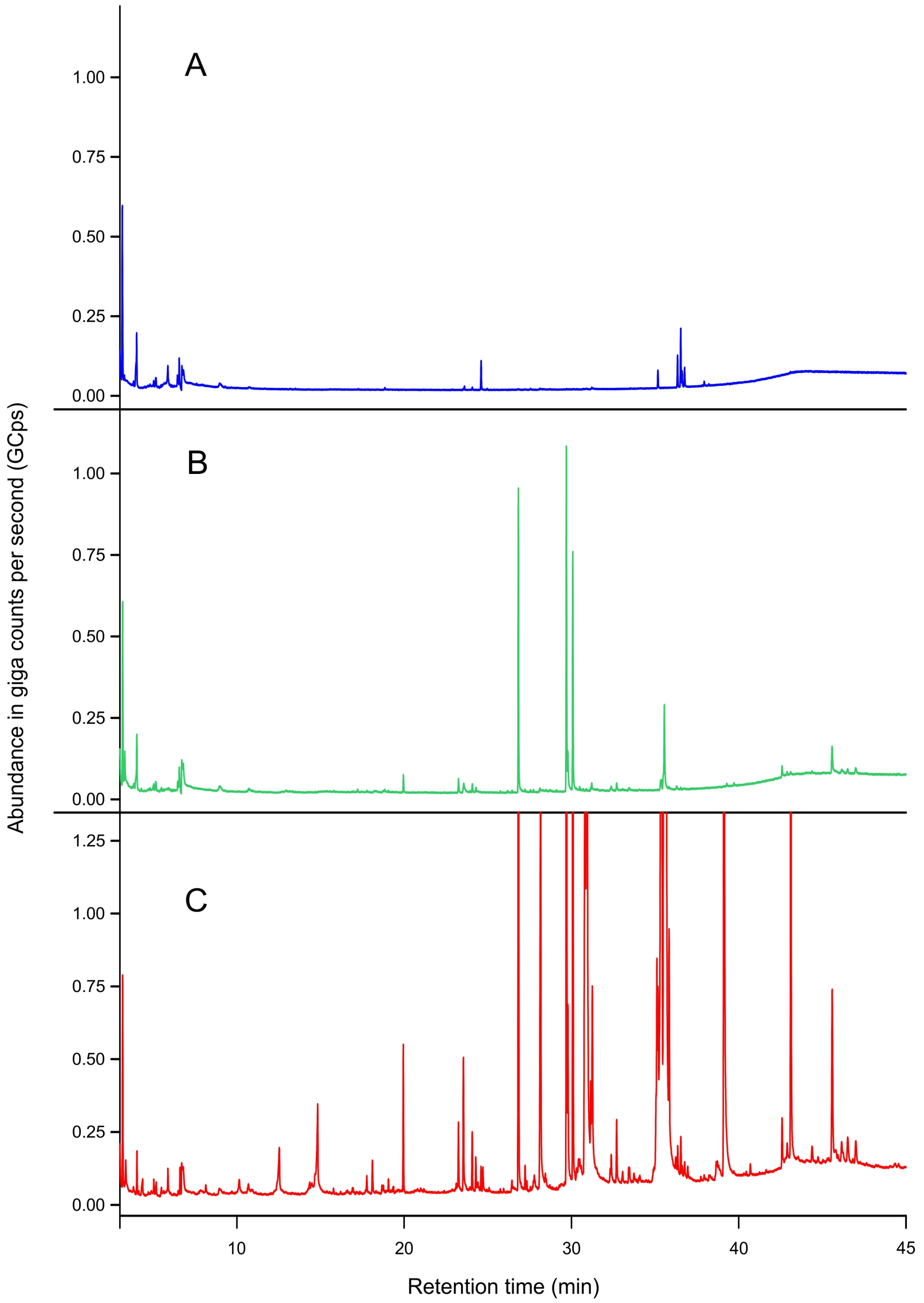

Supplementary Figure 1: Comparison of gas chromatograms of sterile plastic core (blue), sterile cotton after being removed from the plastic core (green), and an actual sample of pedal swab of a brown bear (*Ursus arctos*; red) extracted with methanol. The x-axis is the retention time in minutes and the y-axis is the abundance in giga counts per second (GCps) scale.

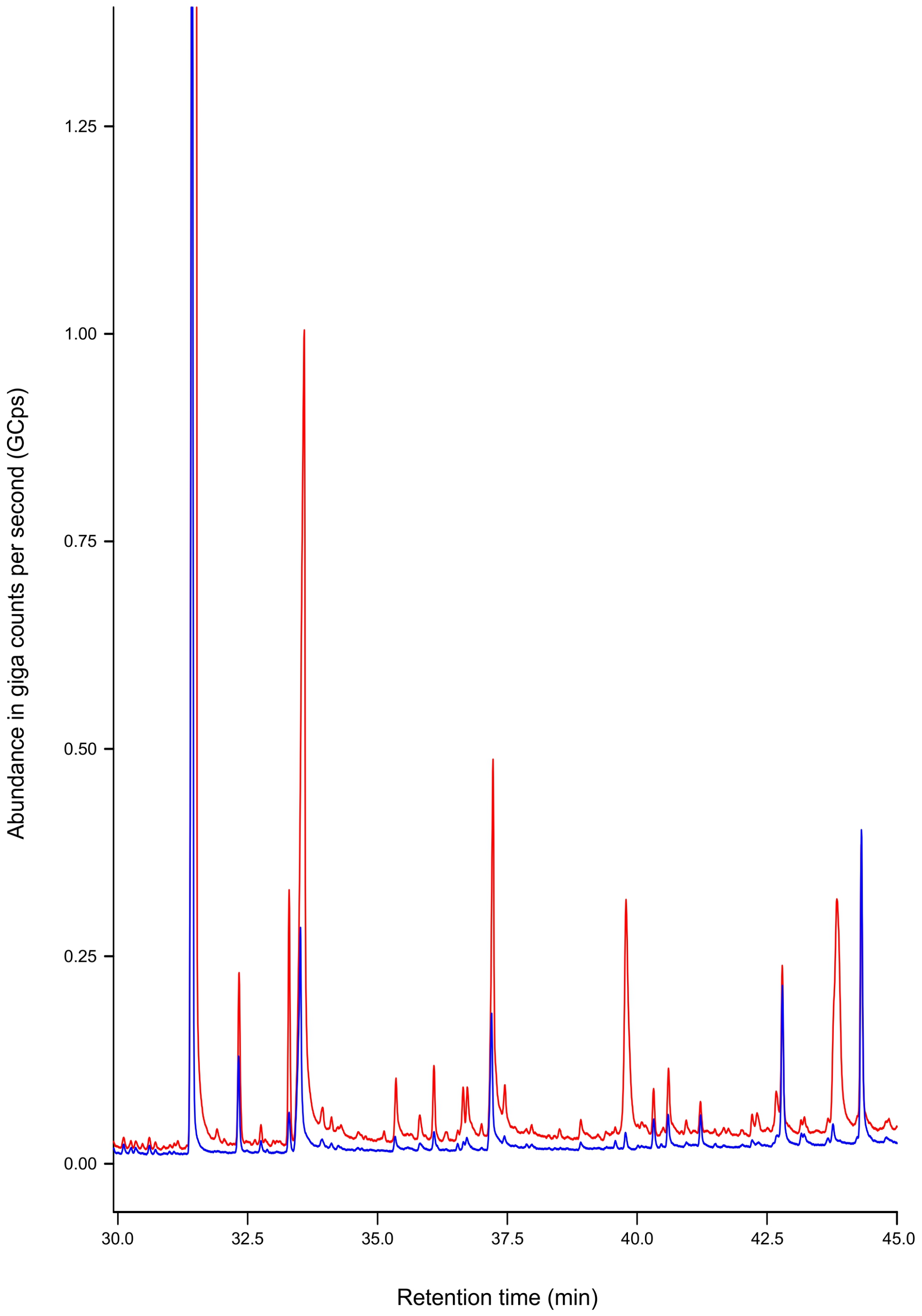

Supplementary Figure 2: Gas chromatograms of pedal swab of a brown bear (*Ursus arctos*) comparing the intensity of the signals obtained with hexane (red) and methanol (blue) extractions. The x-axis is the retention time in minutes and the y-axis is the abundance in giga counts per second (GCps) scale.

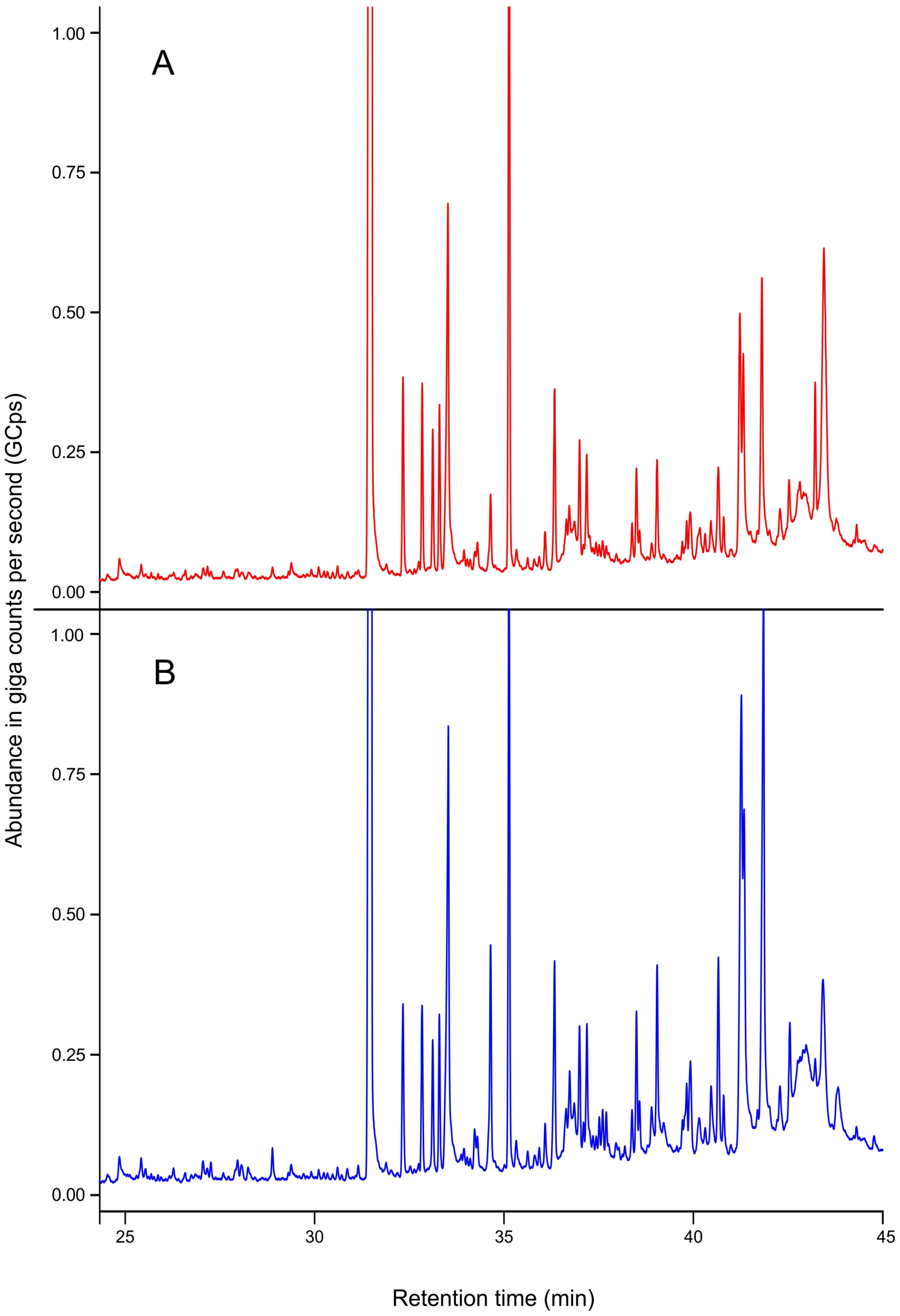

Supplementary Figure 3: Gas chromatograms of tested pedal swab of a brown bear (*Ursus arctos*), and observed similarity in profile peaks in chromatogram after 2 (red) and 24 (blue) hours of extraction. The x-axis is the retention time in minutes and the y-axis is the abundance in giga counts per second (GCps) scale.

3. Supplementary Table

Supplementary Table 1: Comparison of morphological features of apocrine sweat glands in interdigital, metacarpal and metatarsal regions of the sampled adult and yearling brown bear (*Ursus arctos*) males, and control skin sections of adult male. Control areas of lips and shoulders of the yearling male were not available for the skin sections (NA).

Region sectioned	Number of glands associated with hair follicles (range)		Number of segments in one gland (range)		Ratio of hair follicles with apocrine sweat glands to follicles without the glands		Number of sections used for comparison	
	Adult	Yearling	Adult	Yearling	Adult	Yearling	Adult	Yearling
Interdigital	1-4	1-3	10-30	3-19	2:1	2:1	5	8
Metacarpal/ metatarsal	1-3	1-2	5-28	3-20	2:1	2:1	4	14
Lip	1-3	NA	5-22	NA	1:0	NA	5	NA
Left shoulder	1	NA	2-5	NA	1:2	NA	4	NA
